# Effect of Hypoxia-Induced Micro-RNAs Expression on Oncogenesis

**DOI:** 10.3390/ijms23116294

**Published:** 2022-06-04

**Authors:** Giorgia Moriondo, Giulia Scioscia, Piera Soccio, Pasquale Tondo, Cosimo Carlo De Pace, Roberto Sabato, Maria Pia Foschino Barbaro, Donato Lacedonia

**Affiliations:** 1 Department of Medical and Surgical Sciences, University of Foggia, 71122 Foggia, Italy; giorgia.moriondo@unifg.it (G.M.); giulia.scioscia@unifg.it (G.S.); piera.soccio@unifg.it (P.S.); cosimo.depace@unifg.it (C.C.D.P.); mariapia.foschino@unifg.it (M.P.F.B.); donato.lacedonia@unifg.it (D.L.); 2 Institute of Respiratory Diseases, “Policlinico Riuniti” University Hospital of Foggia, 71122 Foggia, Italy; robsabato@libero.it

**Keywords:** microRNA, biomarkers, colorectal cancer, hypoxia, hypoxia-regulated microRNAs

## Abstract

MicroRNAs (miRNAs) are small non-coding RNAs that negatively regulate gene expression at the post-transcriptional level. An aberrant regulation of gene expression by miRNAs is associated with numerous diseases, including cancer. MiRNAs expression can be influenced by various stimuli, among which hypoxia; however, the effects of different types of continuous hypoxia (moderate or marked) on miRNAs are still poorly studied. Lately, some hypoxia-inducible miRNAs (HRMs, hypoxia-regulated miRNAs) have been identified. These HRMs are often activated in different types of cancers, suggesting their role in tumorigenesis. The aim of this study was to evaluate changes in miRNAs expression both in moderate continuous hypoxia and marked continuous hypoxia to better understand the possible relationship between hypoxia, miRNAs, and colorectal cancer. We used RT-PCR to detect the miRNAs expression in colorectal cancer cell lines in conditions of moderate and marked continuous hypoxia. The expression of miRNAs was analyzed using a two-way ANOVA test to compare the differential expression of miRNAs among groups. The levels of almost all analyzed miRNAs (miR-21, miR-23b, miR-26a, miR-27b, and miR-145) were greater in moderate hypoxia versus marked hypoxia, except for miR-23b and miR-21. This study identified a series of miRNAs involved in the response to different types of continuous hypoxia (moderate and marked), highlighting that they play a role in the development of cancer. To date, there are no other studies that demonstrate how these two types of continuous hypoxia could be able to activate different molecular pathways that lead to a different expression of specific miRNAs involved in tumorigenesis.

## 1. Introduction

MicroRNAs (miRNAs) are small non-coding RNAs formed by about 18–22 nucleotides, whose main role is to negatively regulate gene expression at the post-transcriptional level. They act by recognizing specific mRNA targets in order to determine their translation degradation or repression [1]. To date, the function of many miRNAs is not known; however, their involvement in numerous physiological and pathological processes has been demonstrated as follows: in fact, they seem to play a role in cell proliferation, apoptosis, and differentiation [2]. An abnormal regulation of gene expression by microRNAs has been associated with the development and progression of numerous diseases, including cancer [3]. The expression of miRNAs can be influenced by various stimuli such as oxidative stress, inflammatory response, and hypoxia [4]. Assessment of miRNA expression changes during hypoxia is critical to understanding the role of miRNAs in many diseases and inflammatory processes. Although some guidelines have been established, there are no absolute partial pressure values of oxygen (pO_2_) to define hypoxia. In general, pO_2_ of 5% or less in cellular systems indicates hypoxia. Levels of pO_2_ of between 5 and 2% correspond to a condition of moderate hypoxia, while levels <2% represent a condition of marked hypoxia [5].

Hypoxia can be continuous or intermittent; in the latter case, oxygen concentrations alternate between basal and low O_2_ levels. In fact, intermittent hypoxia is caused by a series of repeated episodes of hypoxia and reoxygenation (in vivo, the classic model of intermittent hypoxemia occurs in the course of obstructive sleep apnea), while continuous hypoxia is characterized by constantly low oxygen levels. The pathogenetic mechanisms underlying these two types of hypoxia are completely different [4], and, in this study, we decided to focus our attention on continuous hypoxia.

There are the following two types of continuous hypoxia: moderate and marked. In recent years, some hypoxia-inducible microRNAs (HRMs, hypoxia-regulated microRNAs) have been identified. These HRMs are often activated in different types of cancers, such as breast and colon, suggesting their role in tumorigenesis [6]. Evaluating changes in miRNA expression during hypoxia is critical to understanding the role of miRNAs in many diseases, such as cancer and numerous inflammatory processes. To date, the mechanisms that regulate gene expression during hypoxia are not entirely clear; however, we know that many miRNAs are involved in the development of cancer. In general, it is known that miRNAs are directly involved in the formation of tumors [7], and we also know that about 6% of miRNAs have putative HRE (hypoxia response element) sites in their DNA, regions present in the promoters of hypoxia-inducible genes, indicating these miRNAs as possible targets of HIF-1 (Hypoxia Inducible Factor-1) and suggesting the possibility of their role associated with hypoxia [7]. As the cellular response to hypoxia involves the activation of several transcriptional regulators involved in inflammation, tumor invasion, angiogenesis, cell cycle block, and apoptosis, we believe that a better understanding of all these closely related mechanisms, as well as the identification of miRNAs sensitive to hypoxia, may prove fruitful in the search for new therapeutic targets and in the search for new and more effective anti-tumor therapies.

Based on the previous considerations, in this study we evaluated the expression of different miRNAs in conditions of continuous hypoxia, moderate (2% of oxygen) and marked (0.5% of oxygen), to study the possible relationship between these two types of continuous hypoxia, miRNA, and cancer and identify the miRNAs involved in carcinogenesis that are susceptible to hypoxia. In detail, we evaluated a group of miRNAs (miR-21, miR-23b, miR-26a, miR-27b, and miR-145), which are induced by a hypoxic environment, to better understand how continuous hypoxia could change their signature and to identify their eventual role in colorectal cancer (CRC), which is one of the most frequent tumors in women and men [8].

CRC is a very common malignant tumor, usually located between the junction of the rectum and sigmoid colon. According to a study conducted by Bray et al., CRC is the second cancer for mortality and the fourth for incidence [9]. The patient survival rate 5 years after diagnosis is approximately 65% [10].

Currently, the only prognostic indicator for CRC is represented by histological analysis, so it is very important to find useful biomarkers for the prognosis and diagnosis of this cancer.

To date, it is known that microRNAs can act as suppressors or promoters of CRC. The miRNAs play a role in CRC proliferation, metastasis, angiogenesis, and apoptosis, as well as play essential roles in various biological processes [11].

However, few studies have been carried out on their role in CRC in hypoxic conditions and, in particular, on the role of HMRs in CRC as potential biomarkers for the prognosis and diagnosis of CRC.

Under hypoxic conditions, cancer cells activate a series of molecular pathways driven by the transcription factor HIF-1 and, in response to these stimuli, modify their phenotype by implementing multiple survival strategies. It is, therefore, essential to identify molecular mediators through which HIF-1 controls tumor progression in order to identify new and specific molecular targets for the treatment of colorectal cancer [12].

## 2. Results

The MTT assay showed that at 2, 4, 8, and 24 h of exposure to the different experimental conditions, Caco-2 viability did not change as compared with the control. The survival rate was the same under all tested conditions (data not shown).

**Figure 1:**
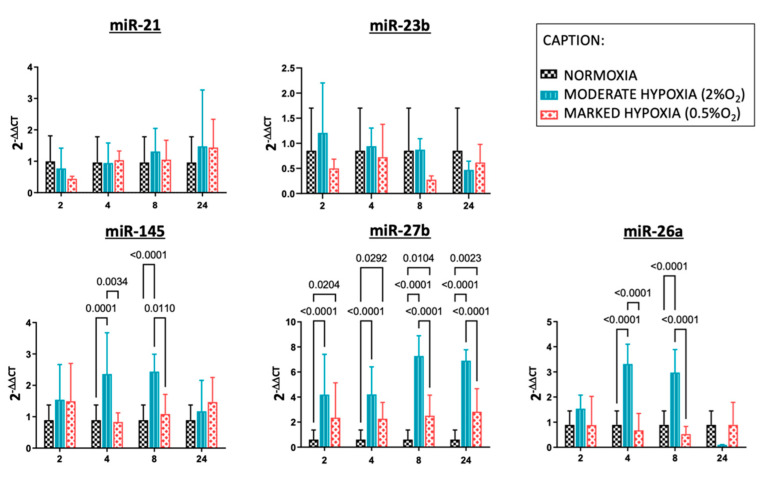
Expression of different miRNAs in normoxia, moderate continuous hypoxia (2% O_2_) and marked continuous hypoxia (0.5% O_2_). Quantitative real-time PCR analysis of differentially expressed microRNAs (miRNAs) in Caco-2 cells in condition of moderate (light blu) and marked (red) continuous hypoxia and normoxia (black). RNU-6B was used as endogenous control. The x axis shows the different times of exposure to hypoxia while the y axis shows the expression of each miRNA. Data represent the mean ± SD.

The expression of miRNAs was different in continuous hypoxia (moderate and marked) and normoxia (Figure 1).

The levels of nearly all analyzed miRNAs were greater in moderate hypoxia versus marked hypoxia.

Notably, miR-145 and miR-26a showed higher levels of expression in moderate hypoxia than in marked one at 4 and 8 h, while for miR-27b, we proved this difference at 8 and 24 h.

We also detected an up-regulation among moderate hypoxia and normoxia at 4 and 8 h of miR-145 and miR-26a. Moreover, for miR-27b, there was the same up-regulation at all analyzed times (2, 4, 8, and 24 h).

In addition, miR-27b showed a higher expression at all times in marked hypoxia compared to normoxia.

On the other hand, miR-23b showed a lower expression at all times in marked hypoxia compared to normoxia, but for this result, there is no statistically significant difference, while only for miR-21, there seems to be no difference between moderate and marked hypoxia.

Therefore, the expression of nearly all analyzed miRNAs was greater in moderate hypoxia versus marked hypoxia, except for miR-23b and miR-21.

**Figure 2:**
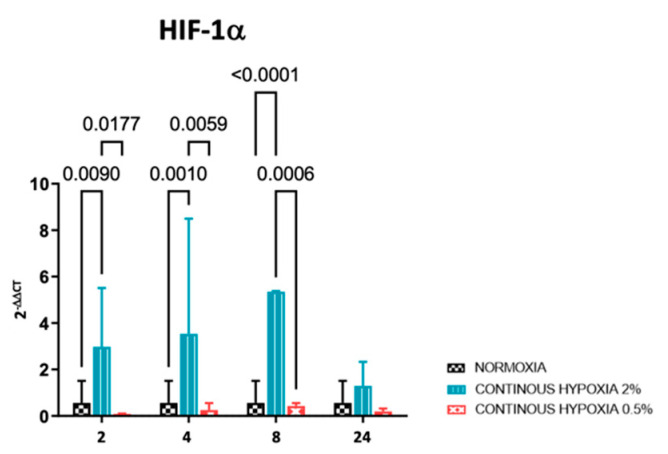
Relative mRNA expression of HIF-1α. Total RNA was extracted, and qRT-PCR was performed in order to quantify HIF-1α. β-actin was used as internal normalizer. Data represent the mean ± SD.

The mRNA expression of HIF-1α either in conditions of normoxia, moderate hypoxia, and marked hypoxia was assessed by qRT-PCR. As shown in Figure 2, the results showed, in agreement with the expression of nearly all analyzed miRNAs, a higher expression of HIF-1α in conditions of moderate hypoxia when compared with normoxia or marked hypoxia. This is true for 2, 4, and 8 h of hypoxia exposure; however, there is no significant expression difference at 24 h. This result could be explained by the fact that cells, under stressful conditions, are able to implement a series of strategies to react to external stimuli.

We also performed a cluster analysis on both moderate and marked continuous hypoxia miRNAs values. However, only for moderate continuous hypoxia do we obtain relevant data.

**Figure 3:**
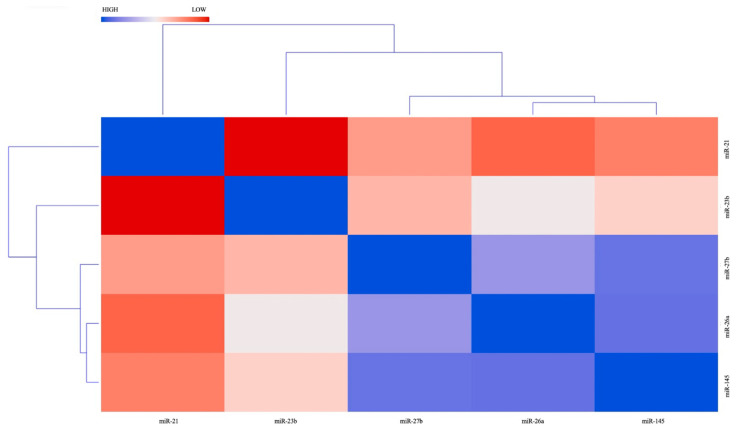
Heat map of miRNAs expression in condition of moderate continuous hypoxia (2% O_2_). The heatmap shows the similarities between the expression profiles of the significantly changed miRNAs in condition of moderate continuous hypoxia. Blue color represents lower than mean intensity and red indicates higher than mean intensity. Each row and each column represent a miRNA.

The differential miRNA expression in the condition of moderate continuous hypoxia (2% O_2_) is shown in the heatmap in Figure 3.

The expression of miRNA was related to each other by means of the cluster analysis, resulting in a negative correlation between miR-21 and miR-23b (Figure 3). Moreover, we found a correlation between miR-26a, miR-27b, and miR-145, which form a cluster.

Generally, miRNAs with similar expression profiles during various experimental conditions are classified into clusters. Thus, this clustering allows the identification of miRNAs involved in the same cellular functions or the same regulatory pathway. Accordingly, the cluster analysis in our study shows a link between three miRNAs (miR-26a, miR-27b, and miR-145) because of their up-regulation during continuous moderate hypoxia (2% O_2_).

## 3. Discussion

We currently know that miRNAs are involved in the development and progression of cancer and that they are able to regulate the expression of many oncogenes and tumor suppressor genes involved in the pathogenesis of cancer [13]. However, it is difficult to fully understand the role of miRNAs in carcinogenesis as their function can vary depending on the target tissue. To date, the molecular mechanisms by which miRNAs modulate cellular processes have yet to be fully elucidated, which is why the study of the specific functions of miRNAs in carcinogenesis could be useful to evaluate their therapeutic potential as diagnostic and prognostic markers of disease [14]. Recently, some studies have identified hypoxia-inducible miRNAs, HRMs, which are often activated in different types of tumors, suggesting their role in tumorigenesis [6]. The present study aimed to evaluate the expression of different miRNAs in conditions of moderate and marked hypoxia to study the possible relationship between these two types of hypoxia, miRNA, and cancer, and to identify the miRNAs involved in carcinogenesis that are susceptible to hypoxia. The main finding of this study is that nearly all of the miRNAs analyzed appear to have increased expression under conditions of moderate hypoxia; however, for miR-145 and miR-26a, this statement is not true when we consider their expression after 24 h of exposure to hypoxia.

In particular, three of them (miR-145, miR-27b, and miR-26a) are more expressed in moderate hypoxia than the marked one. For miR-145 and miR-26a, this is true for 4 and 8 h, whereas for miR-27b, we detected this up-regulation at 8 and 24 h. Vascular endothelial growth factor (VEGF) is a signal protein produced by cells that normally stimulate angiogenesis and that takes part in all those cellular mechanisms that restore normal oxygen supply to tissues following hypoxia. Tumors that over-express VEGF are able to grow and metastasize [15]. HIF-1α is a transcription factor that responds to hypoxia and stimulates the release of VEGF from parts of cells. VEGF binds to receptors on the endothelial cells and triggers a tyrosine kinase signaling pathway that leads to angiogenesis [16]. As previously mentioned, some miRNAs appear to be involved in the regulation of the HIF pathway by acting on specific signaling molecules that function as oncogenes or tumor suppressors. The miRNA most involved in this mechanism is certainly miR-26a [17].

All the miRNAs analyzed in this study are hypoxia-inducible miRNAs, however, there is evidence showing their altered expression in some types of tumors [18]. This, therefore, leads us to hypothesize that hypoxia (moderate or marked) can lead to an alteration of the expression of specific miRNAs involved in the formation of cancer.

MiR-23b is a hypoxia-regulated microRNA involved in apoptosis that appears to be up-regulated in some cancers such as those in the pancreas and colon [6]. In our study, miR-23b showed a lower expression at all times in the marked hypoxia compared to normoxia, but for this result, there is no statistically significant difference. Aberrant expression of miR-23b has been demonstrated in the development of several cancers. Chen L. et al., for example, investigated the oncogenic significance and function of miR-23b in glioma. However, they observed that miR-23b expression was elevated in glioma cells and that miR-23b acted through the HIF-1a/VEGF signaling pathway [19].

MiRNA-21 and miRNA-26a are released from endothelial cells. Previous work has shown that both miRNAs are expressed at the cellular level in response to hypoxic conditions. Therefore hypoxia seems to be a factor capable of inducing the activation of endothelial cells and, consequently, the release of these two miRNAs [20]. Our work shows results consistent with what has just been said. In fact, miR-26a appears higher in moderate hypoxia both compared to normoxia and to marked hypoxia at 4 and 8 h, while miR-21a seems to have a similar expression in marked and moderate hypoxia without any significant statistical difference. For miR-26a, this result is in line with what was previously demonstrated by Lacedonia et al. as far as it is concerned with continuous hypoxia [4].

To date, it is well known that miR-21 is overexpressed in most human tumors, and it promotes malignant growth and progression by acting on multiple targets [21]. In particular, miR-21 induces activation of PTEN (phosphatase and tensin homolog deleted on chromosome 10), AKT (protein kinase B), VEGF, and HIF-1 and consequently tumor progression [22]. Recent data suggest that miR-21 is also involved in promoting inflammation. Indeed, miR-21 appears to be able to reduce the expression of anti-inflammatory molecules such as TGF-β (transforming growth factor-β) [23]. MiR-21 thus constitutes a direct link between tumor-associated inflammatory state and cancer development or progression [24].

Moreover, for miR-26a, many studies have shown that it is dysregulated in various types of cancer [25,26]. Currently, many oncogenes, involved in multiple biological pathways such as proliferation, invasion, differentiation, and angiogenesis, appear to be targets of miR-26a. MiR-26a plays a role in tumorigenesis, acting both as a tumor suppressor and as an oncogene [27].

A recent study by Blick C. et al. showed that miR-145 plays an important role in hypoxia-dependent apoptosis in bladder cancer [28]. In this work, Blick C. and his collaborators demonstrated that miR-145 was significantly increased in response to hypoxia in bladder cancer cells and that this miRNA represents a target gene of HIF. Our work shows high levels of miR-145 in conditions of moderate hypoxia when compared to marked hypoxia or normoxia at 4 and 8 h.

Several studies in the literature suggest that miR-145 is a miRNA that acts as a tumor suppressor by inhibiting tumor growth and angiogenesis, and this miRNA appears to be down-regulated in various types of tumors [29]. Yu Yin et al. observed that miR-145 was significantly downregulated in plasma and tumor tissues of colorectal cancer patients and that miR-145 overexpression inhibited cell proliferation, migration, and invasion. In this previous work, they also demonstrated that miR-145 blocks the activation of the AKT and ERK1/2 pathways and the expression of HIF-1 and VEGF [30]. Based on our results, we, therefore, think that the low levels of miR-145 found in marked hypoxia may play a role in tumor development and progression, unlike when we observe moderate ones.

The same can be said for miR-27b. At present, many studies have reported that miR-27b plays an important role in cancer progression and have shown that miR-27b functions as a tumor suppressor in various types of cancers [31,32]. Chen Y et al. report that miR-27b-3p expression levels were lower than controls in both CRC patients and CRC cell lines. They also demonstrated that miR-27b-3p is capable of inhibiting the proliferation, migration, and invasion of colorectal cancer cells [33].

There are also evidences that miR-27 could facilitate the epithelial-mesenchymal transition (EMT) and the endothelial-mesenchymal transition (EndMT) in several types of cancer, including colorectal cancer through the activation of the transforming growth factor-β (TGF-β) [34,35]. It is well known that the EMT confers several traits to cancer cells that are required for malignant progression and TGF-β is considered to act as a primary inducer of this process [36].

So, based on these considerations and taking into account our results, we can therefore state that low levels of miR-27b in marked hypoxia may have a greater impact on cancer progression than in moderate hypoxia.

## 4. Materials and Methods

### 4.1. Cell Culture and Growth Conditions

Colorectal adenocarcinoma cell lines (CACO-2) were maintained in DMEM (Euroclone, Milan, Italy) supplemented with 10% fetal bovine serum (Euroclone, Milan, Italy), L-glutamine (Euroclone, Milan, Italy), and penicillin/streptomycin (Euroclone, Milan, Italy).

The cells were exposed to continuous hypoxia as follows:

- Moderate continuous hypoxia: we cultured the cells in an incubator (GALAXY 48 R, Eppendorf s.r.l., Milan, Italy) with oxygen maintained at 2% for 2 h, 4 h, 8 h, and 24 h;
- Marked continuous hypoxia: we cultured the cells in an incubator (GALAXY 48 R, Eppendorf s.r.l., Milan, Italy) with oxygen maintained at 0.5% for 2 h, 4 h, 8 h, and 24 h.

For both conditions of hypoxia, normoxic controls were maintained at 37 °C and 5% CO_2_.

### 4.2. Viability Assay

Caco-2 cells (3 × 10^4^ cells/well) were seeded in 96-well plates and exposed to complete medium. Both in normoxia, continuous hypoxia, and marked hypoxia for 2, 4, 8, and 24 h. Cell’s viability was evaluated by MTT (1-(4,5-Dimethylthiazol-2-yl)-3,5-diphenylformazan) according to the manufacturer’s protocol (Sigma-Aldrich, Milan, Italy). The cell’s viability was calculated as follows: %viability = (optical density (OD) _560–655_ of cell/OD _560–655_ of control × 100).

### 4.3. Total RNA Purification and qRT-PCR Analysis

In order to perform miRNA analysis, total RNA was extracted by using TRIzol reagent (Thermo Fisher Scientific, Waltham, MA, USA), according to the manufacturer’s protocol. Concentration and purity of RNA were measured using NanoDrop 1000 Spectrophotometer (Thermo Fisher Scientific). RNA purity was evaluated with the absorbance ratio OD260/OD280. RNA for miRNAs analysis (10 ng) was reverse transcribed into cDNA using TaqMan MicroRNA RT kit (Thermo Fisher Scientific), according to the manufacturer’s protocol. The resulting cDNA transcript was used for detecting miRNA expression by quantitative real-time polymerase chain reaction (qRT-PCR) with Taqman miRNA assay (Thermo Fisher Scientific) according to the manufacturer’s instructions. RNU-6B was used to normalize all RNA samples [37]. The miRNAs expression was calculated using the comparative 2^−ΔΔCt^ method.

Expression of HIF-1 was evaluated by qRT-PCR using SsoAdvanced ™ SYBR^®^ Green Supermix (Bio-Rad, Hercules, CA, USA), as specified by the manufacturer.

Real-time reactions were set up in duplicate for each sample in 96-well plates in a reaction volume of 20 µL containing, respectively, 1X SsoAdvanced ™ SYBR^®^ Green Supermix, 250 nM of specific primers, and 100 ng of cDNA.

The sequences of the primers used for amplification through qRT-PCR were the following:

HIF-1α forward 5′-AAAATCTCATCCAAGAAGCC-3′; HIF-1α reverse 5′-AATGTTCCAATTCCTACTGC-3′; β-actin forward 5′-GACGACATGGAGAAAATCTG-3′; β-actin reverse 5′-ATGATCTGGGTCATCTTCTC-3′.

The reaction was carried out on the ABI-PRISM 7300 instrument according to the manufacturer’s instructions. Gene expression was analyzed according to 2^−ΔΔCt^ relative quantification method using β-actin as internal control.

### 4.4. Statistical Analysis

The results are expressed as mean ± SD. The ANOVA test was used to compare differences among groups. We used the two-way ANOVA test to compare the differential expression levels of miRNAs among moderate hypoxia (2% of oxygen), marked hypoxia (0.5% of oxygen), and normoxia and among different times (2, 4, 8, and 24 h) (GraphPad Software, 7825 Fay Avenue, Suite 230, La Jolla, CA 92037 USA). *p*-value < 0.05 was considered statistically significant.

Clustering analysis was performed on both moderate and marked continuous hypoxia values in order to (i) identify classes of miRNA based on their expression profiles and (ii) analyze the variations in the expression of miRNAs studied.

## 5. Conclusions

In conclusion, with this study, we have identified some miRNAs involved in different ways in the response to different types of continuous hypoxia (moderate or marked), highlighting that the expression of these small molecules can vary under hypoxic conditions and that they could play a role in the development of numerous diseases, including cancer.

Our work showed a change in the gene expression of some hypoxia-inducible miRNAs involved in tumor development and progression. Based on these preliminary results, we believe that moderate and marked hypoxia could activate different molecular pathways according to the severity of the hypoxia; however, further studies are needed in this regard.

To date, according to our knowledge, there are no similar studies in the literature that demonstrate a correlation between hypoxia (moderate and marked), miRNA, and cancer through the identification of a series of miRNAs involved in carcinogenesis that are susceptible to two different types of continuous hypoxia, moderate and marked.

In conclusion, this study demonstrates that continuous hypoxia induces the expression of several miRNAs, some of which appear to be directly involved in cancer formation and progression. Furthermore, it demonstrates how there is a different response between the condition of moderate continuous hypoxia (2%) and that of marked continuous hypoxia (0.5%), bringing to light that the former seems to be, in some cases, much more dangerous in terms of stimulation of the expression of some miRNAs.

## Data Availability

The data presented in this study are available on request from the corresponding author.
